# Supplementary material for: The Association between TRP Channels Expression and Clinicopathological Characteristics of Patients with Pancreatic Adenocarcinoma
Source: Int J Mol Sci. 2022 Aug 12;23(16):9045. doi: 10.3390/ijms23169045 (PMC9408824; doi:10.3390/ijms23169045)
Supplement: Supplementary file 1 [file ijms-23-09045-s001.zip › ijms-1784493-supplementary.pdf]

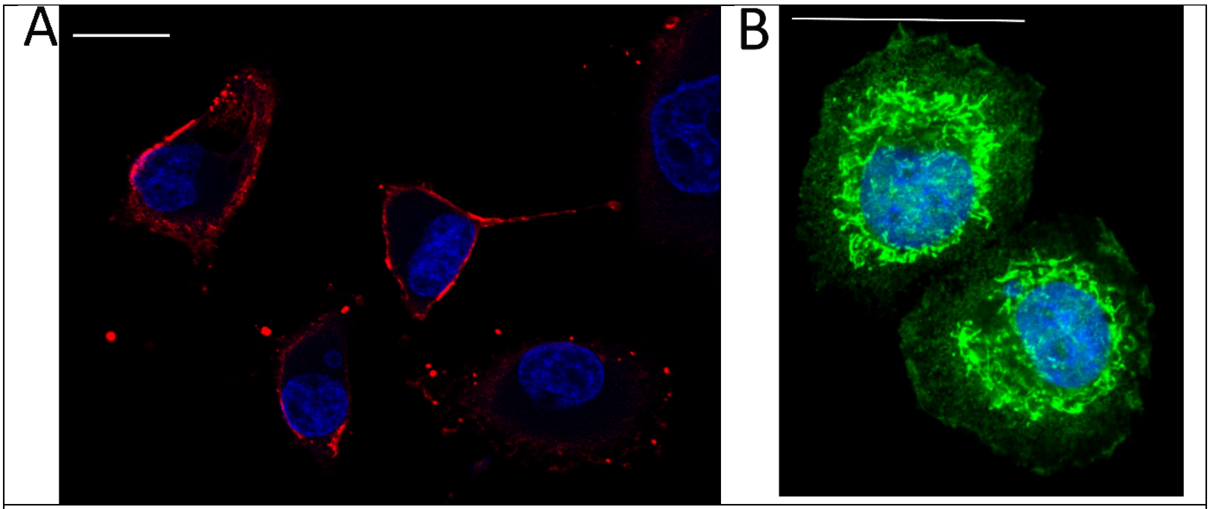

Supplemental Figure S1. Expression of TRPM8 and TRPA1 proteins in Panc-1 cells. A. TRPM8 expression in Panc-1 cells membranes. The cells were incubated with the primary anti-TRPM8 antibody (ab3243; 1:1000) and Alexa 594 conjugated as secondary antibody. The images were collected with a Zeiss Axiovert 200 M microscope fitted with an ApoTome Imaging System. B. Expression of the TRPA1 protein jn Panc-1 cells. Panc-1 cell culture were labelled with primary rabbit polyclonal TRPA1 (PA1-46159, diluted 1:500 in BSA) and Alexa Fluor 488 conjugated goat anti-rabbit secondary antibody (green); nuclei were counterstained with Hoechst 33258 (blue). The images were taken on the Olympus BX53 upright microscope customized for confocal microscopy, magnification 100x, oil immersion. Scales bars represent 10 µm.

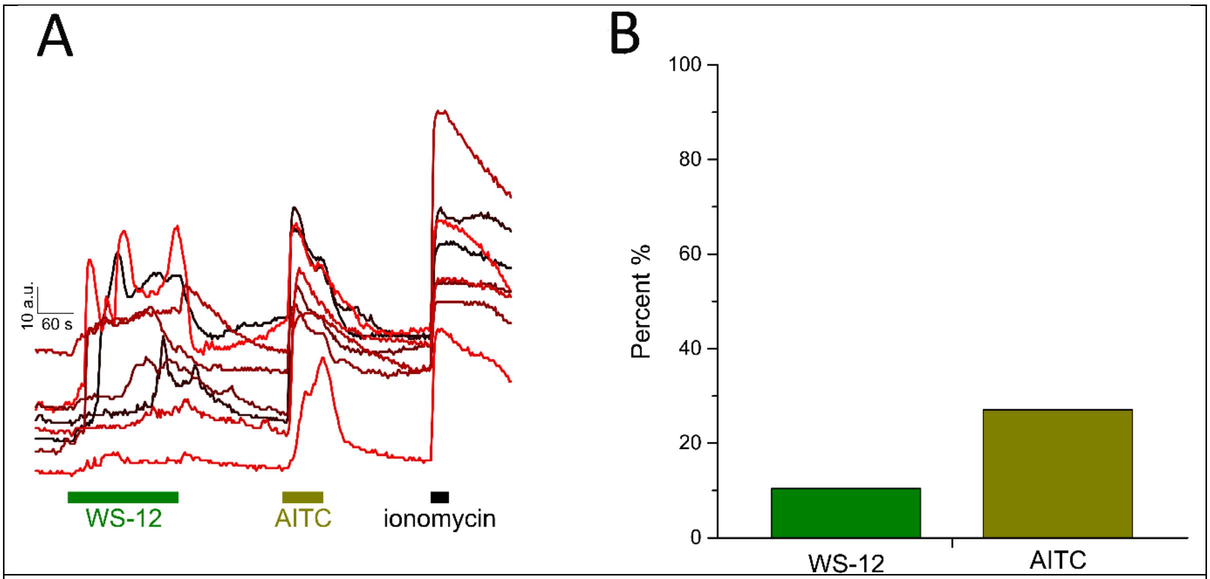

Supplemental Figure S2. Functional expression of TRPM8 and TRPA1 in subpopulations of Panc-1 cells. A. Illustrative examples of fluorescence traces of Panc-1 cells challenged with the TRPM8 agonist, WS-12 (5 µM, 3 min), the TRPA1 agonist, AITC (100 µM, 1 min) and ionomycin (2 µM, 20 s) as control. B. Percentage of Panc-1 cells activated by WS-12 (5 µM) and AITC (100 µM). All tested cells responded to ionomycin (n = 103) while 10.6 % responded to WS-12 (n = 11) and 27.1 % responded to AITC (n = 28).

Immunofluorescence. Cells were plated on 6 wells ibidi μ-Slides. After fixation with paraformaldehyde, the cells were washed 3 times in PBS and permeabilized with 0.2% Triton X-100. After incubation with specific antibodies anti-TRPM8 antibody (ab3243) and anti TRPA1 PA1-46159 and secondary antibodies, images were collected and processed using Adobe Photoshop 5.0 software.

Calcium microfluorimetry

Panc-1 cells were plated on coverslips (25 mm diameter) treated with Poly-D-lysine and incubated in external solution containing 2 μM Calcium Green-1 AM and 0.02 % Pluronic F-127 (Thermo Fisher Scientific) for 30 min at 37 °C. After 30 min of recovery the cells were mounted in a Teflon chamber and illuminated with 470 nm OptoLED light source (Cairn Research). The images were captured through an Olympus IX70 inverted microscope by a CCD camera (Cohu 4910) and analysed with Axon Imaging Workbench 2.2 (Axon Instruments). The standard extracellular solution contained the following (in mM): NaCl, 140; KCl, 4; $MgCl_2$, 1; $CaCl_2$, 2; HEPES, 10; NaOH, 4.54; and glucose, 5 (pH 7.4 at 25 °C). The working solutions were added from stock solutions as follows: allyl isothiocyanate (AITC, Fluka) 100 mM in DMSO, (2S,5R)-2-isopropyl-N-(4-methoxyphenyl)-5-methylcyclohexanecarboximide (WS-12, Sigma) 50 mM in DMSO.
